# Intelligent emission-sensitive routing for plugin hybrid electric vehicles

**DOI:** 10.1186/s40064-016-1802-8

**Published:** 2016-02-29

**Authors:** Zhonghao Sun, Xingshe Zhou

**Affiliations:** School of Computer Science, Northwestern Polytechnical University, Xi’an, China; School of Computer Science, McGill University, Montreal, Canada

**Keywords:** PHEV, Routing, Emission, Navigation

## Abstract

The existing transportation sector creates heavily environmental impacts and is a prime cause for the current climate change. The need to reduce emissions from this sector has stimulated efforts to speed up the application of electric vehicles (EVs). A subset of EVs, called plug-in hybrid electric vehicles (PHEVs), backup batteries with combustion engine, which makes PHEVs have a comparable driving range to conventional vehicles. However, this hybridization comes at a cost of higher emissions than all-electric vehicles. This paper studies the routing problem for PHEVs to minimize emissions. The existing shortest-path based algorithms cannot be applied to solving this problem, because of the several new challenges: (1) an optimal route may contain circles caused by detour for recharging; (2) emissions of PHEVs not only depend on the driving distance, but also depend on the terrain and the state of charge (SOC) of batteries; (3) batteries can harvest energy by regenerative braking, which makes some road segments have negative energy consumption. To address these challenges, this paper proposes a green navigation algorithm (GNA) which finds the optimal strategies: where to go and where to recharge. GNA discretizes the SOC, then makes the PHEV routing problem to satisfy the principle of optimality. Finally, GNA adopts dynamic programming to solve the problem. We evaluate GNA using synthetic maps generated by the delaunay triangulation. The results show that GNA can save more than 10 % energy and reduce 10 % emissions when compared to the shortest path algorithm. We also observe that PHEVs with the battery capacity of 10–15 KWh detour most and nearly no detour when larger than 30 KWh. This observation gives some insights when developing PHEVs.

## Background

Our current transportation system contributes a lot to the climate change due to its heavy emissions. For example, EPA (2015) reported that the US transportation sector produces more than 1.8 billion tons of greenhouse gas in 2011, which shares nearly 30 % of the total emissions in US (UCS 2015). The need to reduce emissions from this sector renews interests in electric transportation and electric vehicles (EVs) come as a promising alternative for conventional vehicles (Al-Alawi and Bradley 2013). In addition, various kinds of marketing models also forecast that there will be a high penetration of EVs in the coming decades (Al-Alawi and Bradley 2013).

A subset of EVs, called plug-in hybrid electric vehicles (PHEVs), are equipped with both electric motor (EM) and internal combustion engine (ICE). PHEVs usually have two operation modes: charge depleting (CD) and charge sustaining (CS) (Zhang and Vahid 2010). When operated at CD mode, PHEVs mainly utilize batteries and operate like all-electric vehicles (Larminie and Lowry 2003). After depleting batteries, PHEVs switch to CS mode and are driven by the ICE like conventional vehicles. By this hybridization, PHEVs get a comparable driving range to conventional vehicles, while become less clean than all-electric vehicles. Current PHEVs usually have a battery with capacity of 2–20 KWh and can be operated at CD mode for only tens of kilometers (Morrow et al. 2008). After this, they will enter CS mode if without recharging batteries, and thus starting to cause much emissions. This paper studies the routing problem that aims at reducing the emissions.

Existing shortest-path based algorithms cannot be applied to this new routing problem, because of the several new challenges: (1) an optimal route may contain circles caused by detour for recharging; (2) PHEVs’ emissions depend not only on the travelling distance, but also on the road slope and the state of charge (SOC) of batteries; (3) batteries can harvest energy by regenerative braking, which makes some road segments have negative energy consumption; (4) it does not satisfy the “principle of optimality” in Ichimori et al. (1981), that is, the sub-route of an optimal route may be not optimal. In this paper, to address these challenges, we propose a green navigation algorithm (GNA) which finds optimal routes: where to go and where to recharge.

To make this problem have the *optimal substructure*, GNA discretizes SOC and introduces the concept of *effective state*. Then the PHEV routing problem is solved by dynamic programming technique. To be specific, we make two major contributions as follows. First, we model maps as augmented directed graphs. Three variables are assigned to each arc: the discretized electricity consumption in CD mode, the gasoline consumption in CD mode and the gasoline consumption in CS mode. The state of the PHEV at a node includes the current SOC and the accumulated emissions. Effective states at a node are those who have minimum emissions at the a SOC level. Then we prove that an optimal route is formed by nodes where the states must be effective. With this optimal substructure, we transform the PHEVs routing problem to one that can be solved by dynamic programming. The time complexity of GNA is polynomial.

We test the GNA on synthetic maps generated by Delaunay triangulation. The results show that routes of GNA can save more than 10 % energy and reduce 10 % emissions when compared to the shortest route. We also observe that the most detours happen when batteries have a capacity of 10–15 KWh and almost no detour happens when larger than 30 KWh. This observation helps on development and deployment of PHEVs. Moreover, our evaluation shows that a denser deployment of recharging facilities helps to reduce up to 20 % emissions additionally. This observation helps on choosing how many charging stations should be built and where to build them.

## Related works

The objective of this paper is finding the emission-optimal strategies for PHEVs between a given source and destination. Thus, on the one hand we present works about energy management of PHEVs, on the other hand we present works about route planning algorithms.

### PHEV energy management

The PHEV energy management problem is to find out the sequence of optimal power split between the internal combustion engine (ICE) and electric motor (EM) at each instant of time that minimizes the fuel consumption (Pisu and Rizzoni 2007). In other words, they aim to minimize the fuel consumption in powertrain level. We summarize the PHEV power management as three stages. The first is non-explicit stage. At this stage, the management strategy does not explicitly seek to optimize energy consumption. The most typical representative is the rule-based control strategies (Baumann et al. 2000; Lin et al. 2003; Schouten et al. 2002; Zhang et al. 2010). These strategies are easier to implement, while the resultant operation may be quite far from optimal due to the omission of detailed dynamic models. The second is explicit-but-suboptimal stage. These kind of strategies (Paganelli et al. 2001, 2002) explicitly formulate a cost function for the fuel consumption to be optimized. An instantaneous minimization on the cost function is carried out. However, without priori information, the instantaneous optimum may be not equal to global optimum. The third is optimal stage. At this stage, global optimal strategies (Gong et al. 2008; Wu et al. 2014; Zhang and Vahidi 2012) integrated with priori information (future driveing cycle, future road conditions etc.) are developed. Obviously, different paths will lead to different emission and fuel consumption. Thus, how to get a better path? This is exactly what we discuss in this paper.

### Route planning algorithm

In addition to these classic shortest-path (SP) algorithms such as Dijkstra, Bellman–Ford and A* etc, some new algorithms have been developed to solve various problems in different backgrounds. Finding the shortest path for a vehicle is originally discussed by Ichimori et al. (1981), where the vehicle has a limited capacity and is allowed to stop and refuel at certain locations. On this basis, Adler et al. (2014) develop a shortest-walk algorithm for electric vehicle (EV) and add a limit to the number of times the EV can exchange batteries. Geisberger et al. (2008) and Sanders and Schultes (2005) propose some hierarchy algorithms which run faster in real road network, but they do not take the various constraints of vehicle into consideration. Moreover, the hierarchy algorithms can not be applied to road networks where the weight a road segment can be negative. Artmeier et al. (2010) propose an energy-optimal routing algorithm for EVs with the constraints of battery capacity and negative weight road resulted from potential energy during deceleration phases. With the same constraints and association, Sachenbacher et al. (2011) develop a more efficient algorithm in the framework of A*. Laporte and Pascoal (2011) develop a labeling algorithm to find a minimum cost path from a source to a destination, along which relay nodes are located at a certain cost, subject to a weight constraint. Brumbaugh-Smith and Shier (1989), Guerriero and Musmanno (2001), Martins (1984) and Skriver and Andersen (2000) also propose similarly labeling algorithms. Different from all these works, we focus on finding an emission-optimal routing for PHEVs. Hausler et al. (2014) provide a stochastic balancing algorithm is presented to reduce the potential for excessively long queues to build up at some charging stations. Different from (Hausler et al. 2014) that manages a fleet of EVs, our paper focus on the routing algorithm for only one EV.

## Methods

This section models the emissions and equivalent fuel consumption (EFC) of PHEVs first. Then the energy splitting strategy used in this paper is described. After that, the model of map is given. Finally, the method based on these models is proposed.

### The model of emissions and EFC

EFC is widely adopted to model the total energy consumption of PHEVs (Johnson et al. 2000; Musardo et al. 2005; Paganelli et al. 2002; Zhang and Vahid 2010). The EFC of a PHEV is the sum of the gasoline consumption and the electricity consumption. In this paper, the electricity consumption is converted to the equivalent gasolines. To be specific, if a PHEV consumes *x* liters of gasoline and *y* KWh electricity, then the EFC is

1 $$\begin{aligned} f_{efc}(x,y)=x+\frac{3.6 * 10^6y}{\mu \rho }, \end{aligned}$$

where $\mu$ is the low heating value of gasoline and $\rho$ is the density.

The emissions of PHEVs are almost totally generated by gasoline combustion, so we use the gasoline consumption to denote the emissions, as shown in (2). Though the emissions from electricity generation are not counted here, they can be easily involved when we get the knowledge about the energy resource of the grid (Paganelli et al. 2002). Adding this part of emissions will not affect the applicability of the proposed algorithm.

2 $$\begin{aligned} f_{emi}(x,y)=x. \end{aligned}$$

### The model of energy splitting

In general, a PHEV can be operated in two modes: charge depleting (CD) and charge sustaining (CS). When the SOC is high, a PHEV can be operated in CD mode (Zhang and Vahid 2010): battery’s charge is depleted to its minimum allowed value with either all-electric operation or blended operation of the EM and ICE (Axsen and Kurani 2008). Otherwise, when the SOC is near its minimum value (*SOC*$_{min}$), the PHEV will be switched to CS mode by blended operation of the ICE and the EM. In CS mode, the battery’s SOC is maintained almost unchanged and the power of the PHEV almost totally comes from the ICE. In this paper, we assume that as long as the SOC is higher than *SOC*$_{min}$, the PHEV will be operated in CD mode; otherwise, it will be switched to CS mode. Different driving modes will lead to different emissions and EFC.

For a road segment $\widehat{ab}$, we use $f_{cd}^e(\widehat{ab})$ and $f_{cd}^g(\widehat{ab})$ to denote the electricity consumption and gasoline consumption in CD mode. $f_{cs}^g(\widehat{ab})$ denotes the gasoline consumption in CS mode. The electricity consumption in CS mode is negligible, so we ignore it. According to the level of SOC, the driving mode on a road can be CD, CS or their blend. Thus, the real emissions and EFC on $\widehat{ab}$ can be formulated as follows.

1. If *SOC*$_a\ge f_{cd}^e(\widehat{ab})$, the PEHV is operated in CD mode. Then
  $$\begin{aligned}&Emission_{\widehat{ab}}=f_{cd}^g(\widehat{ab}), \\&SOC_b=min(SOC_a-f_{cd}^e(\widehat{ab}), SOC_{max}), \\&EFC_{\widehat{ab}}=f_{efc}(f_{cd}^g(\widehat{ab}),SOC_a-SOC_b). \end{aligned}$$
  $SOC_{max}$ denotes the battery capacity.
2. If $SOC_a<SOC_{min}$, the PHEV is operated in CS mode. Then
  $$\begin{aligned}&Emission_{\widehat{ab}}=f_{cs}^g(\widehat{ab}), \\&SOC_b=SOC_a, \\&EFC_{\widehat{ab}}=f_{efc}(f_{cs}^g(\widehat{ab}),0). \end{aligned}$$
3. Otherwise, the PHEV will be operated in CD mode first. When the electricity is depleted, it is switched to CS mode. Then
  $$\begin{aligned}&Emission_{\widehat{ab}}=f_{cs}^g(\widehat{ab})+\frac{SOC_a-SOC_{min}}{f_{cd}^e(\widehat{ab})}\left( f_{cd}^g(\widehat{ab})-f_{cs}^g(\widehat{ab})\right) , \\&SOC_b=SOC_{min},\\&EFC_{\widehat{ab}}=f_{efc}(Emission_{\widehat{ab}},SOC_a-SOC_{min}). \end{aligned}$$

For example, let $SOC_a=4$, $SOC_{min}=0$, $f_{cd}^e(\widehat{ab})=6$, $f_{cd}^g(\widehat{ab})=1$ and $f_{cs}^g(\widehat{ab})=7$. In this case, the SOC is not enough to keep full CD mode along $\widehat{ab}$. So the PHEV will pass through $\frac{4}{6}$ distance of $\widehat{ab}$ after the electricity is depleted and the gasoline consumption along this distance is $\frac{4}{6}*1$. Then the gasoline consumption in the remaining distance is $\frac{2}{6}*7$. The real electricity consumption on $\widehat{ab}$ is 4 and the real gasoline consumption on $\widehat{ab}$ is $\frac{4}{6}*1+\frac{2}{6}*7$.

### The model of maps

A map is modeled as a directed graph $G=(\mathscr {V},\mathscr {C},\mathscr {E})$ where $\mathscr {V}=\{1,\ldots ,n\}$ is the set of transit nodes, $\mathscr {C}\subseteq \mathscr {V}$ denotes the set of recharging nodes where PHEVs can be recharged, $\mathscr {E}=\{1,\ldots ,m\}$ is the set of road segments. A road $\widehat{v_iv_j}\in \mathscr {E}$($v_i,v_j\in \mathscr {V}$) is assigned with three parameters: the CD mode electricity consumption $f_{cd}^e(\widehat{v_iv_j})\in \mathbb {Z}$, the CD mode gasoline consumption $f_{cd}^g(\widehat{v_iv_j})\in \mathbb {R}^+_0$ and the CS mode gasoline consumption $f_{cs}^g(\widehat{v_iv_j})\in \mathbb {R}^+_0$.

**Fig. 1:**
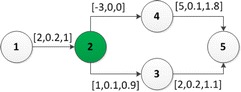
An example of the map model. The numerics near each an arc are the associated $[f_{cd}^e,f_{cd}^g, f_{cs}^g]$ respectively. In this example, $\mathscr {V}=\{1,2,3,4,5\}$, $\mathscr {C}=\{2\}$, $\mathscr {E}=\{\widehat{12},\widehat{23},\widehat{24},\widehat{35},\widehat{45}\}$. $SOC_{max}=4$. $SOC_{1}=3$

Given a source *s* and and destination *d*, a strategy from *s* to *d* is the integration of the route and the charging decisions on this route. A charging operation at $v_i\in \mathscr {C}$ is denoted by $\overline{v_i}$. We assume that the battery will be to fully charged by a charging operation. For example, in Fig. 1, $p=\langle 1,2,4,5\rangle$ and $p'=\langle 1,\overline{2},4,5\rangle$ are two different strategies from 1 to 5. The emissions of *p* and $p'$ are different because of their different charging decisions at the node 2.

In this model, the emissions of a strategy $p=\langle v_1,v_2,\ldots ,v_l\rangle$ can be calculated as

3 $$\begin{aligned} Emission_p=\sum _{i=1}^l{ Emission_{\widehat{v_iv_{i+1}}}}. \end{aligned}$$

The objective of GNA is to find a strategy from the set $\mathscr {P}$ of all possible strategies, which has the minimum emissions.

### The green navigation algorithm

**Fig. 2:**
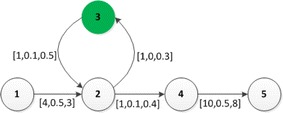
An example to illustrate the challenges of PHEVs navigation. Here $SOC_{max}=10$ and the initial SOC is 4

Before proposing GNA, we have to address the following challenges, which exclude straightforward application of existing algorithms:

1. The optimal strategy may contain circles caused by detours for recharging. As an example in Fig. 2, the optimal strategy from 1 to 5 is $\langle 1,2,\overline{3},2,4,5\rangle$. There is a circle in the route.
2. The “principle of optimal” (Ichimori et al. 1981) is not satisfied in this context. Still as an example in Fig. 2, the optimal strategy from 1 to 5 is $\langle 1,2,\overline{3},2,4,5\rangle$, but its sub-strategy $\langle 1,2,\overline{3},2,4\rangle$ is not the optimal strategy from 1 to 4. The optimal strategy from 1 to 4 is $\langle 1,2,4\rangle$.
3. PHEVs are able to regenerate some electricity when braking or going down slopes. This regeneration indicates that the electricity consumption on some road can be negative, which excludes straightforward application of greedy Dijkstra-like algorithms.
4. The emissions not only depend on the travelling distance, but also depend on the driving modes and SOC. To the best of our knowledge, most algorithms focus on graphs where the weight of arcs are fixed .
5. The battery capacity is limited. This means that additional electricity losses or gains may arise during some roads. For example, if the battery is full before going down a slope, regeneration is no longer possible. Involving the battery capacity constraints into the problem makes it look like a NP-hard problem. Joksch (1966) investigated extensions of the shortest path problem to incorporate such additional constraints. This kind of shortest weight-constrained path problem has been proved to be NP-hard by Michael and David (1979). The similar problems have also been extensively discussed by Beasley and Christofides (1989), Desrochers and Soumis (1988), Handler and Zang (1980), Xiao et al. (2005). However, in our scenario, we will prove that our problem is Pseudo-NP-hard and a global optimal algorithm of polynomial time complexity will be proposed.

First of all, the definitions of **track** and **effective state** are proposed.

#### Track

The track of a strategy is constituted by a sequence of states indexed by integers. A state is a quintuple $\langle Loc,Ele,Emi,Efc,Pre\rangle$ where $Loc \in \mathscr {V}$ is the current location of the PHEV, *Ele* is the current SOC at *Loc*, *Emi* is accumulated emissions from the source to *Loc*, *Efc* is the accumulated EFC from the source to *Loc*, and *Pre* is the index of its direct preceding state.

As an example in Fig. 1, $p=\langle 1,2,4,5\rangle$ and $p'=\langle 1,\overline{2},4,5\rangle$ are two different strategies from 1 to 5. The initial state is $S_0\langle 1,3,0,0,NA\rangle$. The track of *p* is $S_0\rightarrow S_1\langle 2,1,0.2,f_{efc}(0.2,2),S_0\rangle \rightarrow S_2\langle 4,4,0.2,f_{efc}(0.2,-1),S_1\rangle \rightarrow S_3\langle 5,0,0.76,f_{efc}(0.76,3),S_2\rangle$. The track of $p'$ is $S_0\rightarrow S_4\langle 2,4,0.2,f_{efc}(0.2,2),S_0\rangle \rightarrow S_5\langle 4,4,0.2,f_{efc}(0.2,2),S_4\rangle \rightarrow S_6\langle 5,0,0.76, f_{efc}(0.76,6),S_5\rangle$. Although there is no recharging operation in *p*, the emissions of *p* and $p'$ are the same. What’s more, *p* is more energy-effective than $p'$. Because the PHEV has no idle capacity to store the regenerated electricity on $\widehat{2~4}$, since the battery has been fully recharged at 2. This indicates that more charging is not always better, less charging is not always worse.

**Fig. 3:**
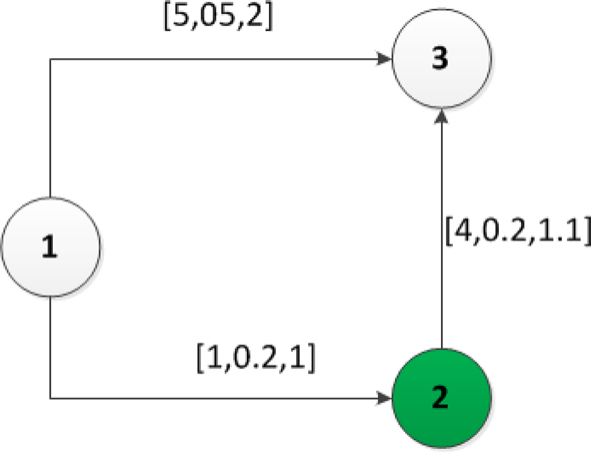
An example to illustrate effective states. The source is node 1 and $SOC_{1}=1$. $SOC_{max}=4$

#### Effective state

Given two states $S_{p_1}$ and $S_{p_2}$, we say $S_{p_1}$ is better than $S_{p_2}$ iff $S_{p_1}.Loc=S_{p_2}.Loc$, $S_{p_1}.Ele=S_{p_2}.Ele$ and $S_{p_1}.Emi<S_{p_2}.Emi$. If there is no state better than $S_{p_1}$, then $S_{p_1}$ is an effective state. As an example in Fig. 3, the initial state is $S_0\langle 1,1,0,0,NA\rangle$. Then the strategies $q=\langle 1,3\rangle$ and $q'=\langle 1,\overline{2},3\rangle$ will generate two different states at the node 3, i.e. $S_3\langle 3,0,1.7,f_{efc}(1.7,1),S_0\rangle$ and $S_{3'}\langle 3,0,0.4,f_{efc}(0.4,5),S_{\overline{2}}\rangle$. $S_{3'}$ is better than $S_3$. In fact, according to the definition of effective state, $S_{3'}$ is an effective state.

##### **Theorem 1**

*For any optimal strategy, its track is formed by effective states only.*

##### *Proof*

Let $p=\langle v_1,v_2,\ldots ,v_t\rangle$ to be the optimal strategy from $v_1$ to $v_t$ and its track to be $S_{v1}\rightarrow S_{v2}\rightarrow \cdots \rightarrow S_{vt}$. Now we prove this theorem by contradiction. Suppose there is a state $S_{vi}$ in the track is not effective. Then according to the definition of effective state, there must exist a sub-strategy $q'$ from $V_1$ to $v_i$ that will lead to a state $S_{vi'}$ better than $S_{vi}$. Then we have $S_{vi'}.Ele=S_{vi}.Ele$ and $S_{vi'}.Emi<S_{vi}.Emi$. Replacing the sub-strategy from $v_1$ to $v_i$ in *p* by $q'$, we can form another strategy $p^*$. This new strategy has fewer emissions than *p*, because

1. *p* and $p^*$, they have same emissions from $v_i$ to $v_t$, because of the same sub-strategy from $v_i$ ta $v_t$ and the same SOC at $v_i$.
2. For emissions from $v_1$ to $v_i$, we have shown that $p^*$ is better than *p*, because $S_{vi'}$ is better than $S_{vi}$. So, when arriving at $v_t$, the emissions of $p^*$ is fewer than *p*. This completes the proof.

$$\square$$

**Fig. 4:**
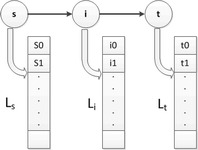
An example of the priority tables. For any node $i\in \mathscr {V}$, it is linked by a priority table $L_i$ to store its current best states. The maximum length of $L_i$ is $SOC_{max}-SOC_{min}+1$

By this result, an optimal strategy can be found by means of state relaxation, i.e. constantly search the effective states and delete the non-effective ones until all states in the priority tables are effective. To be specific, as illustrated in Fig. 4, each node is combined with a priority table to store its currently best states. Then the relaxation of a state is described as follows. For a state $\alpha =\langle i,Ele_i,Emi_i,Efc_i,\beta \rangle$ and for all $\widehat{ij}\in \mathscr {E}$,

1. Generate a new state $\gamma =\langle j,SOC_j,Emi_i+Emission_{\widehat{ij}}, Efc_i+EFC_{\widehat{ij}},\alpha \rangle$. If there is no state in $L_j$ that is better than $\gamma$, insert $\gamma$ to $L_j$ and delete the states worse than $\gamma$ from $L_j$.
2. If *j* is a recharging node, i.e. $j\in \mathscr {C}$, then generate another new state $\delta =\langle j,SOC_{max},Emi_i+Emission_{\widehat{ij}},Efc_i+EFC_{\widehat{ij}},\alpha \rangle$. If there is no state in $L_j$ that is better than $\delta$, insert $\delta$ to $L_j$ and delete the states worse than $\delta$ from $L_j$.

Note that the *insertion* of a new state should be accompanied by a “better” test that compares it with states in the priority table. If there is a states in the priority table that is better than the new one, the new one will be discarded; otherwise it will be stored in the priority table, and all the states worse than this one must be deleted.

The Algorithm 1 summarizes the processes of the GNA. From lines 1 to 4, all the priority tables are set to be empty and the initial state $S_0$ is add to table $L_{source}$. Lines 5–23 iteratively relax all the states until there is no unrelaxed states. After the iterations, we can get the optimal strategy and its track by backtracking from the state of minimum emission in $L_{destination}$.

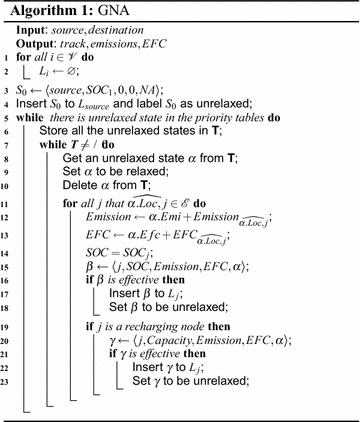



##### **Lemma 1**

*Let node 1 be the source node and denote the SOC at node 1 by*$SOC_1$. *Then, the initial state*$S_0\langle 1,SOC_1,0,0,NA\rangle$*is an effective state*.

##### *Proof*

If $S_0$ is not effective, this indicates that there is a circle from node 1 to node 1 while the gasoline consumption on this circle is negative. Obviously, this is impossible. $\square$

##### **Theorem 2**

*The length of an optimal strategy (or track) is less than*$(SOC_{max}-SOC_{min}+1)*n$.

##### *Proof*

In our model, we have discretized all the possible SOC to a finite set $\{SOC_{min},SOC_{min}+1,\ldots ,SOC_{max}\}$. According to the definition of effective state, the maximum number of the effective states of a node are $SOC_{max}-SOC_{min}+1$. Thus, there are $(SOC_{max}-SOC_{min}+1)*n$ effective states at most. Suppose there is a track longer than $(SOC_{max}-SOC_{min}+1)*n$, then all these states should be effective according to Theorem 1. Thus, in this case, there are at least two states in the track are the same according to the *pigeon hole principle* (Trybulec 1990), i.e. exist a subtrack $\cdots S_x\rightarrow S_{x+1}\rightarrow \cdots \rightarrow S_y\cdots$ where $S_x=S_y$. Deleting the subtrack $S_{x+1}\rightarrow \cdots \rightarrow S_y$ from the original track, the remaining track is still an optimal track. Thus, we prove that the length of an optimal path is less than $(SOC_{max}-SOC_{min}+1)*n$. $\square$

##### **Theorem 3**

*Before finding the optimal strategy (or track), GNA generates at least one effective state in a iteration (Lines 6–23).*

##### *Proof*

Assume there is an optimal track *T*1: $S_0\rightarrow S_1\rightarrow \cdots \rightarrow S_t$, then all the states in this track are effective according to Theorem 1. In Algorithm 1, we relax all the unrelaxed states in all priority tables at each iteration. Now we prove it through induction:

1. Before the first iteration, $S_0$ is effective according to Lemma 1. After this iteration, we relaxed $S_0$ and generate all its successor states including $S_1$. $S_1$ is effective because of $S_1 \in T1$.
2. Consequently, before the *i*th ($i<t$) iteration, we assume $S_{i-1}$ ($S_{i-1} \in T1$) is effective. We relax $S_{i-1}$ and generate all its successor states including $S_i$. $S_i$ is effective because of $S_i \in T1$.

$$\square$$

##### **Theorem 4**

After up to $(SOC_{max}-SOC_{min}+1)*n$ iterations, GNA can generate all effective states. The time complexity of GNA is $O(SOC_{max}^2n^2)$ .

##### *Proof*

According to Theorem 3, GNA generates at least one effective state in an iterate. Moreover, we have proved in Theorem 2 that the maximum number of effective states of all nodes are less than $(SOC_{max}-SOC_{min}+1)*n$. So, after up to $(SOC_{max}-SOC_{min}+1)*n$ iterations, GNA can generate all effective states. For each iteration, GNA relaxes $(SOC_{max}-SOC_{min}+1)*n$ states at most, so the total time complexity of GNA is $O(SOC_{max}^2n^2)$. $\square$

Thus far, we complete the proof of the optimality and the time complexity of GNA.

**Fig. 5:**
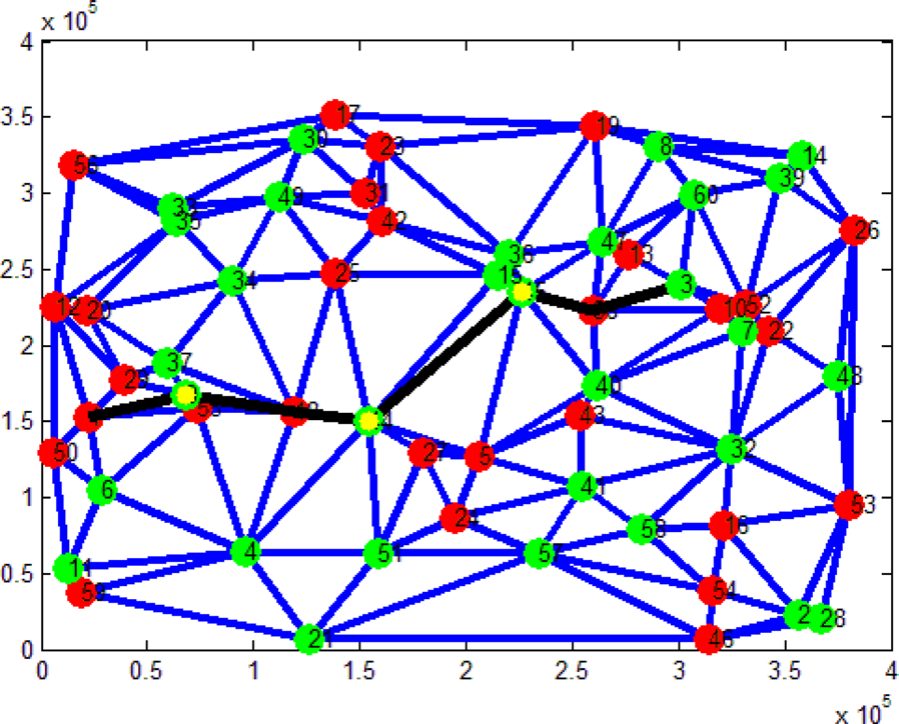
An example of the generated map. The *red nodes* are normal nodes, the *green nodes* are recharging nodes, the *bold black line* is the path, the *green and yellow nodes* on the path represent recharging operations

## The result and discussion

### Experiment setting

We test GNA on synthetic datas. The directed graphs are generated according to strategy presented in Adler et al. (2014). To be specific, we randomly generate the set of nodes $\mathscr {V}$ in a plane. The set of arcs $\mathscr {E}$ is defined as the Delaunay triangulation of these nodes, the length of an arc is set to be the sum of the Euclidean distance and a positive random number. Then we assign a random altitude to each node. Finally, the electricity consumption and gasoline consumption of different driving mode are given out by the road distance, altitude variation, coefficient of friction etc. by referring Larminie and Lowry (2003) and Sachenbacher et al. (2011). The recharging nodes in the graph are randomly selected from $\mathscr {V}$ with a given probability. An example of the generated map is shown in Fig. 5.

To evaluate the performance of GNA, we compare it with two other algorithms: the Bellman–Ford algorithm for the shortest path and EFC-optimal algorithm. Bellman–ord algorithm is a famous shortest-path algorithm which is able to deal with graphs with negative-weight arcs. The EFC-optimal algorithm is same as GNA but the objective is minimizing the EFC. We set the density of the recharging nodes to be 50 %, i.e. randomly select 50 % nodes from $\mathscr {V}$ as the recharging nodes. The capacity of battery is set to be 5 KWh. The SOC of the battery is divided into 51 equal portions, i.e. 0, .1, .2, ldots, ($SOC_{max}=50,SOC_{min}=0$).

### Comparison of emissions

**Fig. 6:**
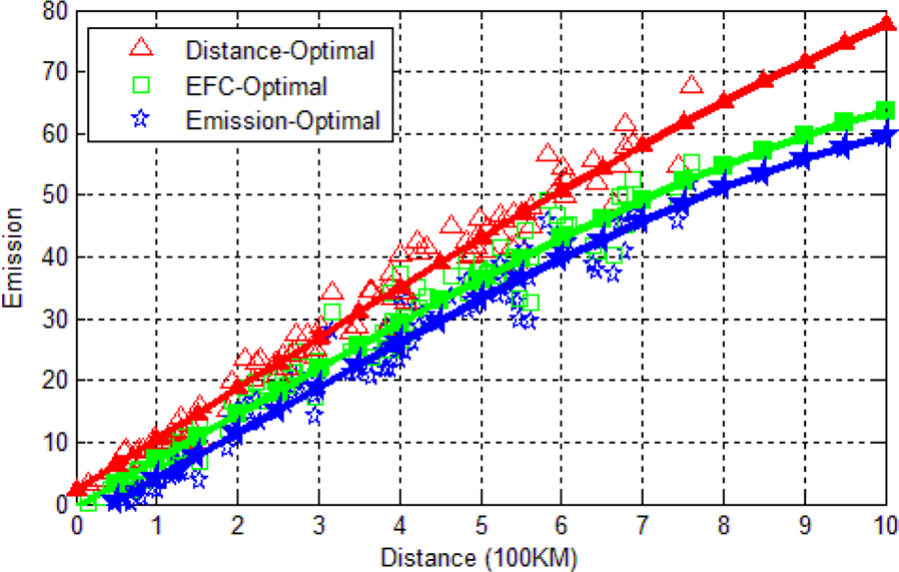
Emissions on various distance. One point in the figure represents the result of a test. The three trajectories are obtained from curve fitting of corresponding points with the *same color*. The following figures are also illustrated in this form

In a test, we generate a map and a pair of source and destination, then we run the three algorithms once on this data. The test is repeated 300 times and we record the emissions and the corresponding distance of the paths. The record data is illustrated in Fig. 6. Figure 6 shows that with the increase of distance, the emissions of the three algorithms increase approximately linearly. The emissions of GNA is slightly better than the EFC-optimal algorithm. By adopting GNA, more than 10 % emissions can be reduced compared to the shortest path by Bellman–Ford algorithm.

### Comparison of EFC

**Fig. 7:**
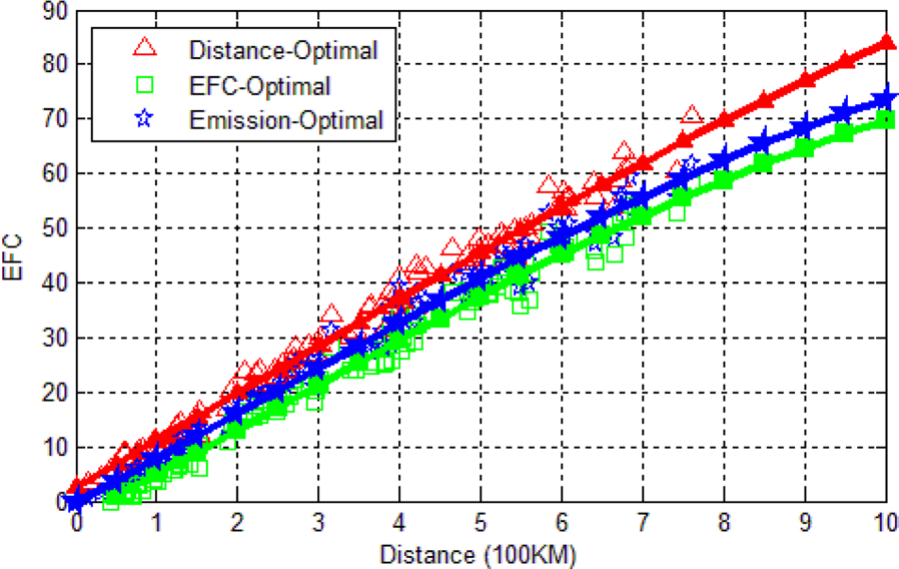
EFC on various distance

In addition to emissions, we also test the energy efficiency of GNA. In each test, we still generate a map and a pair of source and destination, then we run the three algorithms once on this data. The test is repeated 300 times and we record the EFC and the corresponding distance of the paths. The record data is illustrated in Fig. 7. Obviously, both GNA and the EFC-optimal algorithm are more energy-effective than Bellman–Ford algorithm. The EFC of EFC-optimal algorithm is slightly better than GNA. By adopting GNA, more than 10 % EFC can be reduced, compared to the shortest path by Bellman–Ford algorithm.

### Comparison of detouring rate

**Fig. 8:**
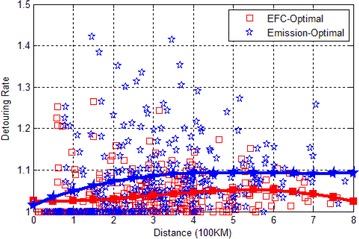
Detouring rate

In GNA, to reduce emissions, it is inevitable to detour to recharge the battery. In each test, we generate a map and a pair of source and destination, then we run the three algorithms once on this data. After each test, we record the length of the paths resulted from the three algorithms. We use $\frac{Distance\quad of\quad GNA}{Distance\quad of\quad Bellman{-}Ford}$ and $\frac{Distance\quad of\quad EFC-optimal}{Distance\quad of\quad Bellman{-}Ford}$ to indicate the detouring rate. We repeat the test 300 times and the test results are shown in Fig. 8.

We can conclude from the figure that the average detouring rate is ${<}1.1$, i.e. the path resulted from GNA is just 10 % longer than Bellman–Ford algorithm. However, in some cases, the paths by GNA is 40 % longer than the shortest paths by Bellman–Ford algorithm. We can also observe that the EFC-optimal algorithm is more distance-effective than GNA.

### Comparison of running time

We have proved that the time complexity of GNA is $O(SOC_{max}^2n^2)$. As we all know, the time complexity of Bellman–Ford algorithm is $O(n^3)$. Because the principle of GNA and EFC-optimal algorithm are the same, so we do not test the EFC-optimal algorithm here. The hardware platform of this test is “Intel Pentium 4 3.2GHZ Dual-Core CPU + 2GBRAM”. The software platform is “Windows 7 Professional X64+Matlab 2012b”.

**Fig. 9:**
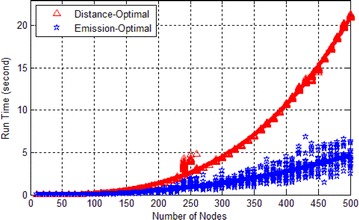
Running time

In the tests, we set the scale of $|\mathscr {V}|$ to be 10, 20, ..., 500 respectively. In each scale, we run the two algorithms 20 times. In each running, we generate a map of the preset scale and a pair of source and destination, then we run the two algorithms once on this data. We record the running time of each test and the result is shown in Fig. 9.

In Fig. 9, Bellman–Ford algorithm runs faster than GNA when $n<50$, but the GNA runs faster than Bellmaaalgorithm when $n>50$. This result exactly verify our proof of GNA’s time complexity. Specifically, in our test, the $SOC_{max}$ is 50, the time complexity of GNA and Bellman–Ford algorithm are $O(SOC_{max}^2n^2)$ and $O(n^3)$. Because the $SOC_{max}$ is fixed, so the running time of Bellman–Ford algorithm will outpace GNA with the increasing of N sooner or later.

### Impact of the battery capacity

In the above tests, we set the battery capacity to be 5 KWh, but now we will quantitatively analyze the capacity’s impact on emissions, energy consumption and detouring rate.

**Fig. 10:**
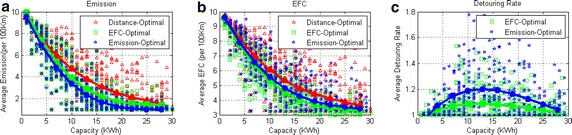
The evaluations of the battery capacity. In the figures, charging node density is 50 %. **a** The variation of average emissions with the increasing of battery capacity. **b** The variation of average EFC with the increasing of battery capacity. **c** The variation of average detouring rate with the increasing of battery capacity

In the test, we set the battery capacity to be 1, 2, ..., 30 KWh. The charging node density is fixed at 50 %. Then we run the three algorithms 20 times on each preset capacity. The result is shown in Fig. 10. We can conclude from the figure that (1) extending the battery capacity from 1 to 30 KWh, all the algorithms can reduce more than 80 % emissions and 60 % energy consumption, (2) increasing the capacity can not always reduce detouring rate, (3) when the capacity is larger than 30 KWh, both the emissions and detouring rate are approximately optimal, (4) when the capacity is between 5–25 KWh, the benefit of GNA is significant; coincidentally, most of today’s PHEVs’ capacity falls in this range.

When the capacity is between 1 and 13 KWh, increasing the capacity enhance the detouring rate actually. This is because, when the capacity is too small, the emission from detour will cover the emission reduction from this recharge. In this case, with the increasing of capacity, some previously uneconomical detour will become worthwhile. Then, with the continuing increasing of capacity, the detouring rate begins to decline. In this case, due to the capacity is large enough, the recharging times on a route can be reduced thus reduce the detouring rate.

### Impact of charging station density

Intuitively, more charging opportunities will lead to more emission reduction and energy savings. It also seems that more charging opportunities can reduce the unnecessary detours. Unfortunately, the truth is not always the case.

**Fig. 11:**
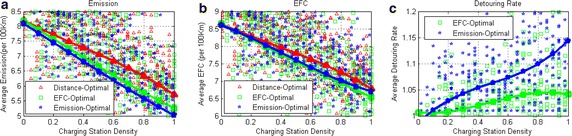
The evaluations of the charging station density. In the figures, the battery capacity is 5 KWh and $SOC_{max}=50$. **a** The variation of average emissions with the increasing of charging station density. **b** The variation of average EFC with the increasing of charging station density. **c** The variation of average detouring rate with the increasing of charging station density

In this part of test, we set the charging station density to be 4, 8, 12,..., 100 %. In each density, we run the three algorithms 200 times. We record the emissions, EFC and detouring rate of each test. The results are shown in Fig. 11.

The Fig. 11a shows that increasing the charging facility deployment could reduce more than 40 % emissions whatever algorithms adopted. The Fig. 11b shows that increasing the charging facility deployment could reduce more than 30 % energy consumption. However, in Fig. 11c, increasing charging facility deployment can not reduce the detouring rate. Instead, it increases the detouring rate slightly. Because in this part of test, we set the capacity to be 5 KWh. This capacity is so small that most of the road segments in the map exceed its range. Thus, in a map with sparse recharging nodes, a detour for recharging may be too far while the electricity recharged is just a little. In this cases, the detours happen relatively less. While in a map with dense recharging nodes, the probability of cost-efficient detours is relatively higher. To drive in CD mode as much as possible, PHEVs have to select route with shorter road segments, thus increasing the detouring rate. To further verify this issue, we set the capacity to be 30 KWh and test the detouring rate again. The result shows that the detouring rate will decline when increasing the recharging node density. This observation helps on the the construction of charging stations and the development of PHEVs.

## Conclusion

This paper proposes a green navigation algorithm for PHEVs that focus on emission minimization. GNA addresses problems that exclude straightforward application of existing shortest-path based algorithms: (1) an optimal route may contain circles caused by detour for recharging; (2) PHEVs’ emissions depend on not only the travelling distance, but also on the road slope and the state of charge (SOC); (3) batteries can harvest energy by regenerative braking, which makes some road segments have negative energy consumption. Then we prove the optimality of GNA and show that its time complexity is $O(SOC_{max}^2n^2)$. We evaluate GNA on synthetic data. The results show that routes by GNA can save more than 10 % energy and reduce 10 % emission, compared to the shortest route. We also observe that the most detours happen when batteries have a capacity of 10–15 KWh and nearly no detour when larger than 30 KWh. Moreover, we observe that dense deployment of recharging facilities helps to reduce more than 20 % emissions additionally. These insights are significant to the deployment of recharging stations and development of PHEVs. For the future work, we will improve GNA by adding limitation to the detouring rate and we will test our algorithm on a real map.
